# Cathepsin-B and cathepsin-L expression levels do not correlate with sensitivity of tumour cells to TNF-*α*-mediated apoptosis

**DOI:** 10.1038/sj.bjc.6601297

**Published:** 2003-10-14

**Authors:** A Gewies, S Grimm

**Affiliations:** 1 Max-Planck-Institute for Biochemistry, Am Klopferspitz 18a, 82152 Martinsried, Germany

**Keywords:** cell death, cathepsin, overexpression, lysosomes

## Abstract

Recently, evidence has been accumulated that besides the caspase proteases, lysosomal cathepsins may play a role in apoptosis induction. This is especially significant as many human tumour cells express high levels of cathepsins, which might sensitise these cells to specific proapoptotic stimuli mediated by cathepsins. We found that TNF-*α*-mediated DNA fragmentation in tumour cells was significantly reduced in the presence of E64d and CA074Me, two inhibitors of lysosomal cysteine proteases. Transient transfection of cathepsin-B (Cath-B) and -L (Cath-L) resulting in expression levels comparable to those found in many tumours did not sensitise tumour cells to TNF-*α*-mediated apoptosis. As lysosomal proteases are thought to be activated by their release from this organelle into the cytosol, we used the lysosomotropic detergent *N*-dodecyl-imidazole-HCl (NDI-HCl) to disturb lysosomal integrity efficiently and trigger the release of its proteolytic content into the cytosol. Treatment of HeLa cells with NDI-HCl resulted in cell death, which, however, could also not be influenced by augmented Cath-B or -L expression levels. Therefore, our data do not support the hypothesis that the high Cath-B or -L expression levels frequently detected in tumour cells might be exploited to target selectively those tumours for an enhanced cell death effect induced by lysosomotropic agents.

The expression levels of the lysosomal papain-like cysteine proteases cathepsin-B (Cath-B) and cathepsin-L (Cath-L) are frequently strongly elevated in tumours of diverse origin. Their secreted forms are thought to contribute to cancer progression and metastasis either by directly degrading the extracellular matrix or by proteolytically activating other extracellular proteases (Chauhan *et al*, 1991; Duffy, 1996; Kos and Lah, 1998; Yan *et al*, 1998; Sloane, 1990; Turk *et al*, 2000; Konduri *et al*, 2001). Therefore, Cath-B and Cath-L are usually regarded as tumour markers correlated with unfavourable clinical prognosis. On the other hand, in recent years, evidence has been collected that lysosomal proteases and particularly Cath-B might be involved in the mediation of apoptotic cell death triggered by various stimuli. This hypothesis has been built on observations that, firstly, lysosomal integrity is disturbed during the apoptotic process with concurrent release of the lysosomal content into the cytosol (Guicciardi *et al*, 2000; Foghsgaard *et al*, 2001; Kagedal *et al*, 2001; Mathiasen *et al*, 2001; Neuzil *et al*, 2002). Secondly, cathepsin inhibitors can reduce the response of cells to some apoptosis inducers (Foghsgaard *et al*, 2001; Kagedal *et al*, 2001; Katz *et al*, 2001; Kiso *et al*, 2001; Li *et al*, 2001; Mathiasen *et al*, 2001). Thirdly, cells deficient or downregulated in Cath-B are more resistant to TNF-mediated apoptosis (Guicciardi *et al*, 2000; Foghsgaard *et al*, 2001). Fourthly, *in vitro* cathepsins can trigger cytochrome *c* release from the mitochondria into the cytosol (Guicciardi *et al*, 2000; Stoka *et al*, 2001). This and the fact that tumours frequently contain high levels of cysteine proteases led to the hypothesis that Cath-B-like proteases may prove useful in selectively targeting tumour cells for apoptosis induction (Foghsgaard *et al*, 2001).

In this report, we show that TNF-induced cell death in tumour cells could be reduced in the presence of cysteine protease inhibitors E64d and CA074Me, but that overexpression of Cath-B and Cath-L comparable to the levels detected in tumour cells did not increase the apoptosis sensitivity of those cells to TNF. Even the proapoptotic effect of the lysosomotropic detergent *N*-dodecyl-imidazole, which directly causes lysosomal instability, was not affected by the intracellular content of Cath-B or Cath-L. Thus, our data do not support the hypothesis that high levels of cathepsin expression might result in the enhanced response of tumour cells to proapoptotic stimuli.

## MATERIALS AND METHODS

### Materials

All fine chemicals were obtained from Sigma-Aldrich, unless otherwise specified. TNF-*α* was obtained from Biomol, the pan-caspase inhibitor zVAD-fmk was from Enzyme Systems Products, E64d was from Biomol or Peptides International, CA074Me was from Calbiochem or Peptides International, zFR-AMC was from Enzyme Systems Products or Biomol, *N*-dodecyl-imidazole was from Toronto Research Chemicals Inc., and its hydrochloride salt was prepared essentially as given in Dubowchik *et al* (1995).

### Cell culture

HeLa cells and McA-RH7777 cells (kindly provided by Professor Dr GM Kostner, Graz) were cultured in DME medium (Sigma) supplemented with 2 mM L-glutamine, 1 mM pyruvate, 100 U ml^−1^ penicillin+100 *μ*g ml^−1^ streptomycin or 100 *μ*g ml^−1^ gentamycin and 10% FCS. PC3 cells were kept in RPMI medium (Gibco) containing the same supplements as described above for the DME medium. Cells were incubated at 37°C at 5% CO_2_.

### Constructs

Coding sequences of hCath-L (Accession M20496), hCath-B (Accession L16510), and hCath-D (Accession M11233) were obtained by RT–PCR from human cDNA and were cloned into the pcDNA3Δ vector, which was derived from the pcDNA3 vector (Invitrogen) by deletion of the neomycin resistance region. The obtained cDNA sequences were sequenced and compared to the above given Genbank sequence entries. For stable transfection experiments, the corresponding constructs were subcloned into the complete pcDNA3 vector containing the neomycin resistance cassette.

### Transfections

HeLa cells and McA RH 7777 cells were transfected using the Effectene transfection reagent (Qiagen). For this, 0.2 *μ*g plasmid DNA together with 50 ng pEGFP (Clontech) were diluted in 100 *μ*l of EC-Buffer, 1.6 *μ*l enhancer was added, and after 5 min of incubation at RT 2 *μ*l of the effectene transfection reagent was added, followed by 15 min of incubation at RT. The transfection mix was given to HeLa cells in a six-well plate, which prior to that had been washed with PBS and then 2 ml of fresh DME medium (10% FCS) had been added. The medium was changed 10 h post-transfection. This protocol could be up- and downscaled for different culture formats. The transfection efficiency could be controlled by fluorescence of the co-transfected pEGFP and was determined by FACS analysis.

### PI-FACS analysis

Apoptosis detection by sub-G1 DNA content was performed by propidium iodide FACS analysis as described (Mund *et al*, 2003).

### MTT assay

Cells were transfected in 10-cm dishes as described; 24 h later cells were harvested and reseeded in equal numbers (approximately 7500 cells/well) in the wells of 96-well plates. At 24 h after reseeding, cells were treated as indicated in a total volume of 100 *μ*l DME medium. At the time of analysis, MTT assays were essentially performed according to Mosmann (1983). ‘Loss of viability, %’ was calculated using the following equation: (*U*−*S*)/U*100, where *U* is the absorption value of the untreated control and *S* is the absorption value of sample.

### Immunoblotting

For detecting protein expression, cells were harvested by trypsinisation, washed with PBS, and lysed in Triton-X buffer (50 mM Tris-HCl pH 8.0, 150 mM NaCl, 1% Triton-X 100) 10 min on ice. After centrifugation at 14 000 **g** in a microfuge, supernatants were obtained as cytoplasmic extracts, which were quantified for protein content using the Bradford reagent (BioRad). Western blotting was performed as described (Bauer *et al*, 1999) using either monoclonal anti-Cath-L antibody (Transduction Laboratories) or monoclonal anti-Cath-B antibody (Ab-2, Oncogene Research Products).

### Cathepsin enzymatic assay

Cells were left untreated or were transfected with the pcDNA3Δ expression vector containing the ORFs of Cath-L, or Cath-B, and at the time of analysis the cathepsin activity assay was performed using zFR-AMC (Enzyme Systems Products) as a substrate, applying a protocol derived from Kamboj *et al* (1993). AMC fluorescence was measured with a Fluoroskan Ascent FL fluorecence plate reader (Labsystems) at a wavelength of 380 nm for excitation and 450 nm for emission. For measuring Michaelis–Menten kinetics, the assay was performed as described above in determining the initial turnover rates of the zFR-AMC substrate at various concentrations (50, 100, 200, 400, and 600 *μ*M) in the presence of 0.5 *μ*g protein extract from Cath-B overexpressing HeLa cells.

### Cell-free activation of caspases for inhibitor experiments with E64d

Cytoplasmic extracts were prepared and activated by the addition of cytochrome *c* and dATP in the absence or presence of various concentrations of E64d, and were analysed by a caspase enzymatic assay essentially as described (Gewies *et al*, 2000).

### Immunocytochemistry

Cells were seeded on cover glasses, and 24 h later were incubated for 16 h in the absence or presence of NDI-HCl, CHX, or TNF/CHX. Immunostaining was carried out as described (Mathiasen *et al*, 2001) using an anti-Cath-L mAb (1 : 100, Transduction Laboratories) and an anti-mouse FITC-conjugated polyclonal Ab (1 : 500, Pharmingen) as the primary or secondary antibody, respectively. Fixed and stained cells were covered with an embedding buffer (0.5 M Tris-HCl pH 8.2, 0.02 g ml^−1^ DTT, 0.04 g ml^−1^ polyvinyl alcohol, 40% glycerol), fluorescence was observed under a fluorescence microscope (Axioscop 2, Zeiss), and pictures were taken with a digital camera (Visitron Systems, Puchheim) using the IPLab imaging software (Spectra Services, NY, USA).

## RESULTS

### Cysteine protease inhibitors E64d and CA074Me suppress TNF-mediated apoptosis

**Figure 1:**
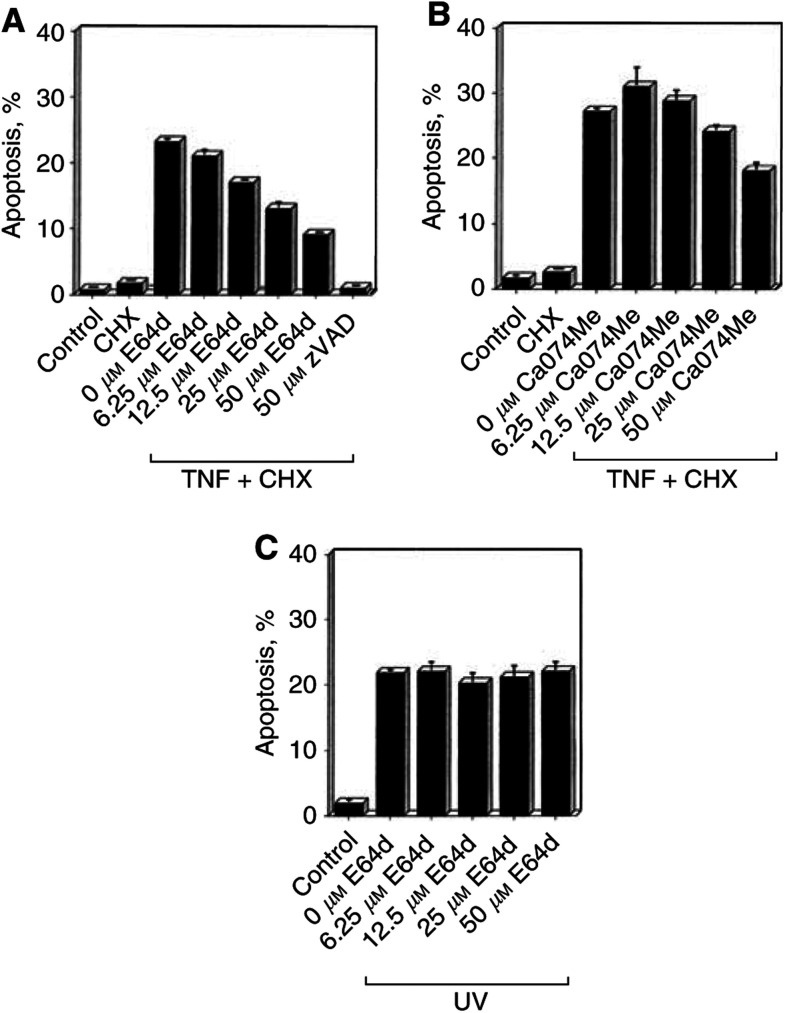
Effect of cathepsin inhibitors on TNF- or UV-induced apoptosis in HeLa cells. Cells were preincubated for 3 h with the indicated amounts of E64d or CA074Me and then cotreated with 10 ng ml^−1^ TNF and 5 *μ*g ml^−1^ CHX (**A, B**) or irradiated with 40 mJ cm^−2^ UV light (**C**). During treatment or after irradiation, respectively, the cells were further kept in the presence of the indicated concentrations of inhibitor and were analysed 12 h (TNF) or 20 h (UV) later by propidium iodide FACS analysis. The values for every data point were acquired in triplicate and are given as the percentage (mean±s.d.) of apoptotic cells with sub-G1 chromosomal DNA content. The data shown are representative for several independent experiments.

**Figure 2:**
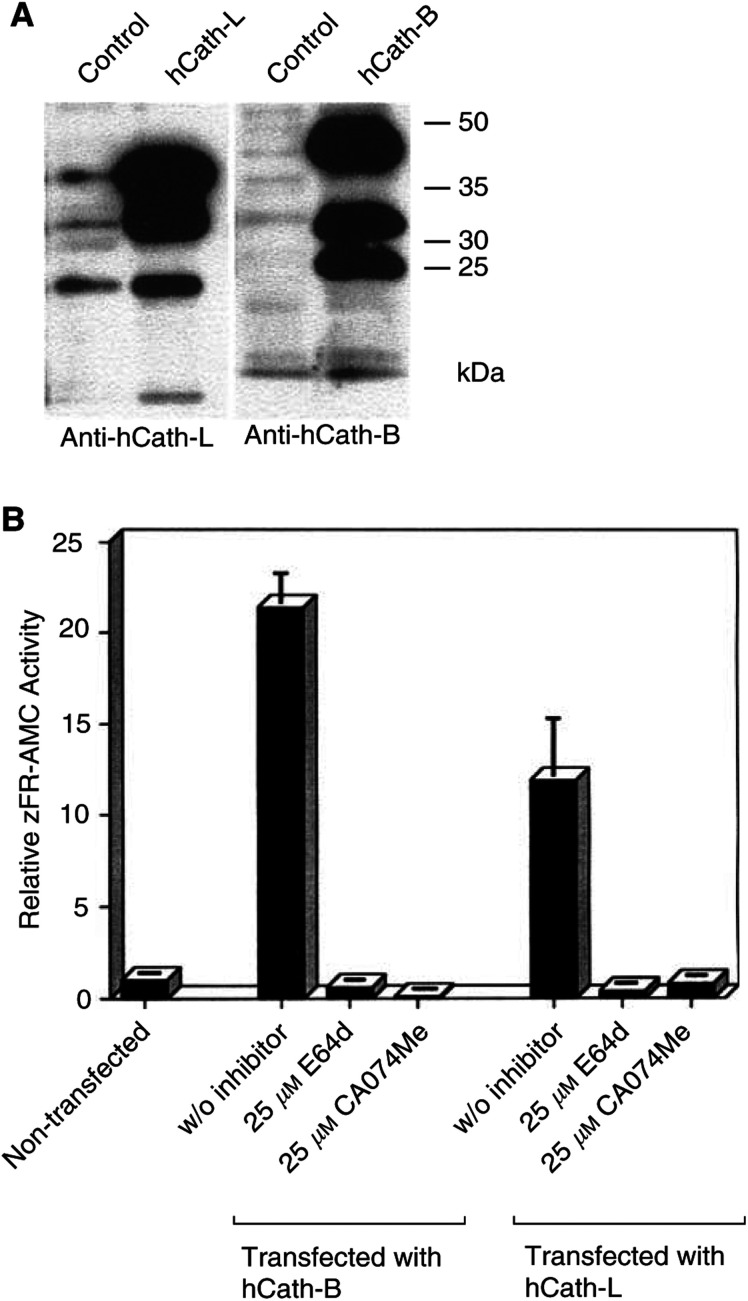
Overexpression of hCath-L and hCath-B in HeLa cells and inhibition of their enzymatic activity by E64d and CA074Me. Expression vectors containing the cDNAs of human cathepsin-L (hCath-L) or human cathepsin-B (hCath-B) were transfected into HeLa cells. (**A**) Cells were harvested 24 h post-transfection, and cell extracts were prepared and analysed for the expression of hCath-L and hCath-B by immunoblot analysis. (**B**) At 24 h post-transfection, cells were incubated for 3 h in the absence or presence of the indicated inhibitor, harvested, and assayed for cathepsin activity. The relative activity units (means±s.d.) were normalised to the activity measured in nontransfected cells. Statistics are as described in Figure 1.

First, we asked whether lysosomal cysteine proteases might be involved in apoptosis induced by TNF in the cervical carcinoma cell line HeLa. Cell death was assessed by DNA fragmentation in HeLa cells cotreated with TNF and cycloheximide (CHX). The pan-caspase inhibitor zVAD-fmk could completely prevent the demise of the cells, indicating an apoptotic mode of cell death (Figure 1A

). This cell death could also be inhibited in the presence of the broad-spectrum cysteine protease inhibitor E64d in a dose-dependent manner, albeit not as efficiently as with zVAD-fmk (Figure 1A). We also used CA074Me, a described specific Cath-B inhibitor (Buttle *et al*, 1992), and likewise detected a decrease of TNF-induced DNA fragmentation (Figure 2B

). Testing the prostatic carcinoma cell line PC3 as a further cell system, we could confirm the inhibitory effect of E64d, CA074Me, and zFA-FMK, a further Cath-B inhibitor (Rasnick, 1985), on TNF-induced DNA fragmentation (data not shown). To address the question of how general the observed inhibitory effect of E64d on apoptotic DNA fragmentation is, we tested its impact on UV-mediated cell death in the HeLa cell system. As shown in Figure 1C, E64d did not inhibit apoptosis induced by UV irradiation under the conditions used.

The observation that the Cath-B inhibitor CA074Me is able to reduce TNF-induced apoptosis in HeLa cells (Figure 1B) suggested an involvement of Cath-B in this cell death process. Consequently, we wanted to prove the specificity of CA074Me on Cath-B activity. To this end, we transiently overexpressed hCath-B or hCath-L in HeLa cells for subsequent inhibitor studies. Immunoblot analysis demonstrated the efficient overexpression of these two cathepsins, whereas endogenous levels of Cath-L and Cath-B in comparison were quite low (Figure 2A). We also measured cathepsin activity in extracts of Cath-B- or Cath-L-transfected cells, which were grown for 3 h in the presence or absence of 25 *μ*M E64d or 25 *μ*M CA074Me, respectively. Concentrations in the range of 25 *μ*M had been used in recent reports (Foghsgaard *et al*, 2001; Varghese *et al*, 2001) and were necessary to elicit an inhibitory effect (Figure 1). As expected, E64d could strongly inhibit both hCath-L and hCath-B enzymatic activity (Figure 2B). Unexpectedly, the supposedly specific Cath-B inhibitor CA074Me not only inhibited the enzymatic activity of the overexpressed hCath-B but also that of the overexpressed hCath-L. However, this observation is in line with a recent report (Montaser *et al*, 2002), which revealed that before cleavage by cellular esterases the methyl ester CA074Me cannot be regarded as a specific Cath-B inhibitor and also affects other lysosomal cysteine proteases such as Cath-L. We also tested the effect of E64d on caspase enzymatic activity *in vitro*: caspase activity induced by the addition of cytochrome *c* and dATP to cell extracts in a cell-free system was not significantly reduced in the presence of 50 *μ*M E64d in the extract (data not shown), therefore excluding the possibility that the effect of E64d on TNF-induced apoptosis is due to the concurrent inhibition of caspases. Thus, Cath-B and also related lysosomal cysteine proteases such as Cath-L should be responsible for mediating the E64d- and CA074Me-sensitive TNF signal.

As shown in Figure 2, the overexpression of hCath-B and hCath-L could be obtained by transient transfection of HeLa cells in terms of both protein levels detected in immunoblots (Figure 2A) as well as cathepsin activities (Figure 2B). In these experiments, we usually observed an increase of cathepsin activity by at least 6- to 10-fold for hCath-L or by 10- to 20-fold for hCath-B when compared to endogenous cathepsin activity. This extent of increased enzymatic activity is comparable to the situation found in malignant human tumour cells for which elevated cathepsin-expression and -activity levels have been described (Chauhan *et al*, 1991; Lah and Kos, 1998; Yan *et al*, 1998).

### Transient overexpression of Cath-D but not of Cath-L and Cath-B induces apoptosis in HeLa cells

Given the possible involvement of Cath-B-like cysteine proteases in TNF-mediated apoptosis in HeLa cells (Figure 1), we decided to investigate the influence of cathepsin expression levels on the sensitivity of HeLa cells to the TNF cell death signal. Especially, Cath-B has been suggested to play an essential role in TNF-mediated apoptosis (Leist and Jaattela, 2001), and has been proposed to represent a therapeutical target in cancer therapy of tumours with elevated levels of cysteine proteases (Foghsgaard *et al*, 2001).

**Figure 3:**
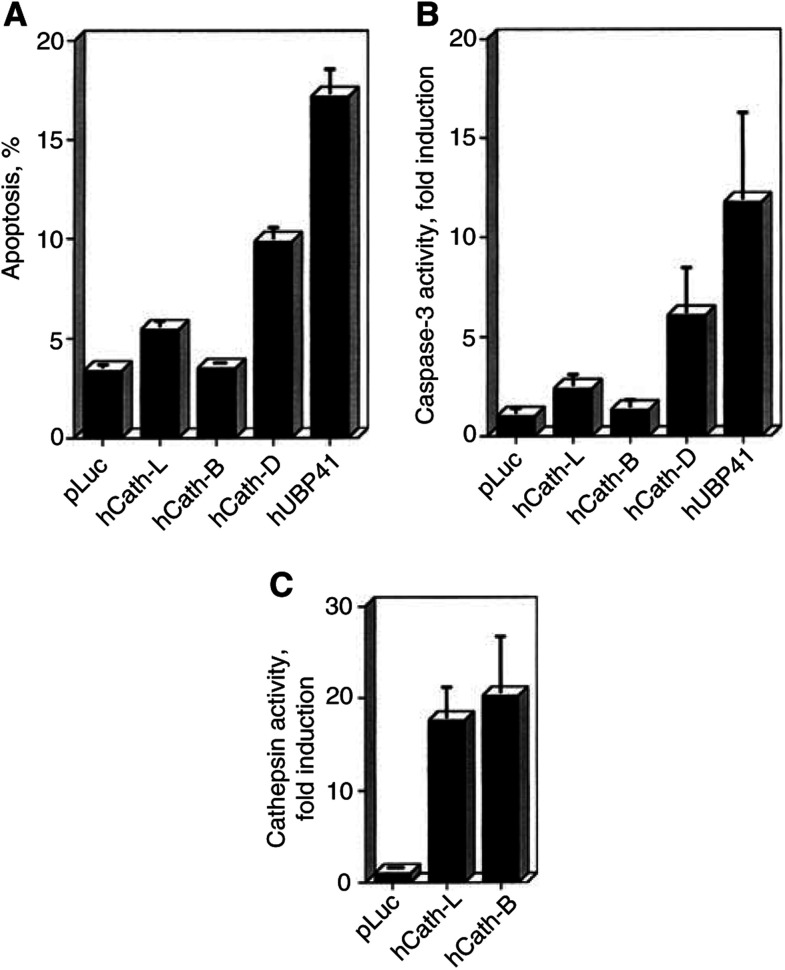
Overexpression of hCath-D, but not of hCath-L or hCath-B results in apoptosis induction in HeLa cells. HeLa cells were transfected with hCath-L, hCath-B, hCath-D, or hUBP41 expression vectors together with a pEGFP expression vector for the determination of transfection efficiency. (**A**) At 48 h post-transfection, cells were harvested for propidium iodide FACS analysis for the detection of apoptotic cells with sub-G1 DNA content. Transfection efficiency was included in the calculation of the percentage of apoptosis. (**B**) At 32 h post-transfection, the transfected cells were harvested for the detection of caspase-3 activity and (**C**) for verification of the overexpression of cathepsins by the zFR-AMC enzymatic activity. Statistics are as given in Figure 1.

We first examined the effect of increased cathepsin expression on the viability of HeLa cells (Figure 3

). We observed cell death in terms of morphology, DNA fragmentation, and caspase-3 enzymatic activity, when an hCath-D expression vector was transiently transfected into HeLa cells (Figures 3A and B). The ectopic overexpression of Cath-D in HeLa cells was previously shown to induce cell death dominantly (Deiss *et al*, 1996). As an additional positive control, we overexpressed the cysteine protease hUBP41, a member of the family of ubiquitin-specific proteases (USPs), which we recently found to possess dominant apoptosis-inducing activity (Gewies and Grimm, 2003). In contrast, the overexpression of hCath-L and hCath-B did not result in significant cell death, even though in the case of hCath-L a slight increase in DNA fragmentation values and caspase-3 activity could be detected (Figures 3A and B), concomitant with an increased number of detached cells (not shown). When we determined the cathepsin activity, we observed an approximately 20-fold increase in these experiments (Figure 3C).

### Augmented levels of hCath-L or hCath-B fail to sensitise HeLa or McA RH 7777 cells to TNF-induced cell death

**Figure 4:**
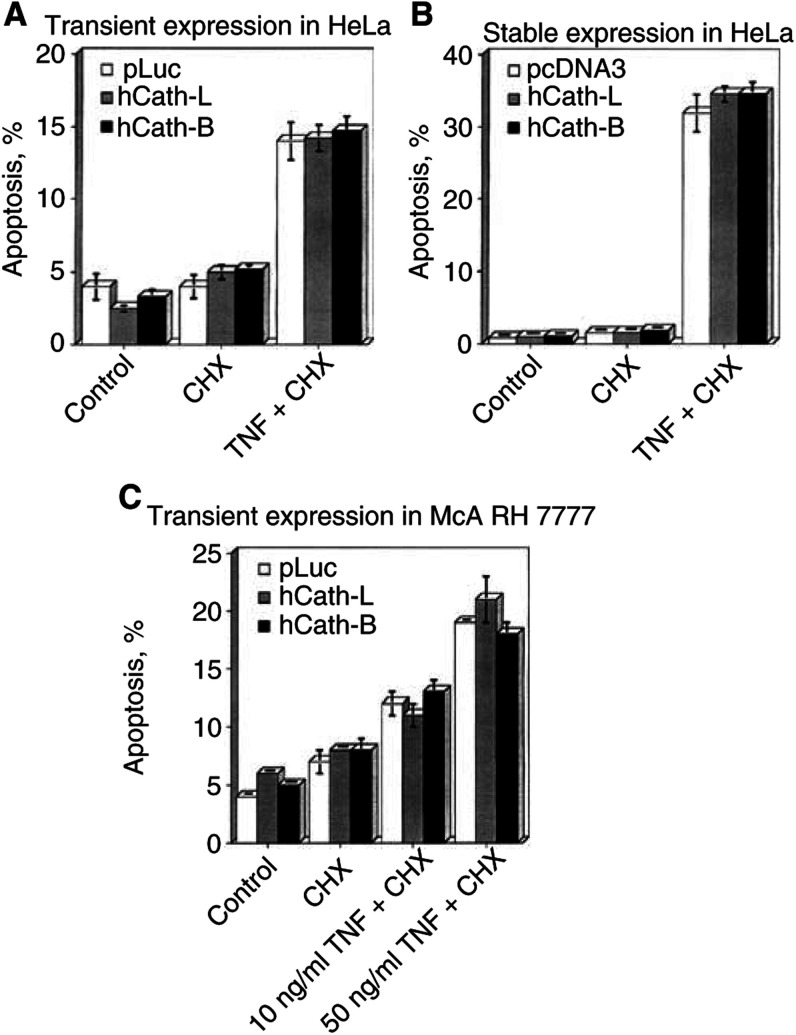
Sensitivity to the TNF-mediated death signal is not enhanced by overexpression of hCath-L or hCath-B. (**A**) HeLa cells were transfected with pLuc, hCath-L, or hCath-B expression vectors, and 36 h post-transfection were incubated in the absence or presence of 5 *μ*g ml^−1^ CHX or 10 ng ml^−1^ TNF +5 *μ*g ml^−1^ CHX. After 10 h of treatment, cells were harvested for propidium iodide FACS analysis. Transfection efficiency in this experiment was 40% as determined by pEGFP cotransfection and EGFP FACS analysis. (**B**) HeLa cell pools selected for stable expression of pcDNA3, hCath-L, or hCath-B were incubated in the absence or presence of 5 *μ*g ml^−1^ CHX or 10 ng ml^−1^ TNF+5 *μ*g ml^−1^ CHX. After 12 h of treatment, cells were harvested for propidium iodide FACS analysis. (**C**) McA RH 7777 rat hepatoma cells were transiently transfected with pLuc, hCath-L, or hCath-B expression vectors, and 36 h post-transfection were incubated in the absence or presence of 1 *μ*g ml^−1^ CHX, 10 ng ml^−1^ TNF+1 *μ*g ml^−1^ CHX, or 50 ng ml^−1^ TNF+1 *μ*g ml^−1^ CHX. After 12 h of treatment, cells were harvested for propidium iodide FACS analysis. Transfection efficiency in this experiment was 40% as determined by pEGFP cotransfection and EGFP FACS analysis. Statistics are as given in Figure 1.

With our results indicating that cathepsins participate in mediating TNF-induced apoptosis (Figure 1), we speculated that elevated cathepsin expression levels might influence the sensitivity of HeLa cells to the apoptosis-inducing signal triggered by TNF. As shown in Figure 4A

, HeLa cells transiently transfected with hCath-L or hCath-B did not show any increased apoptosis when compared to control transfected cells. We also generated HeLa cell pools stably overexpressing hCath-L and hCath-B at levels of two- or four-fold enzymatic activity, respectively, compared to control cells. Also, those HeLa cells did not exhibit an enhanced response to TNF-induced apoptosis (Figure 4B). Since Cath-B has been reported to play a major role in the apoptosis of hepatocytes (Roberts *et al*, 1999; Guicciardi *et al*, 2000,), we also transiently overexpressed hCath-L or hCath-B in the rat hepatoma cell line McA RH 7777. The treatment of these McA RH 7777 cells with TNF did not yield any significant differences in apoptosis levels between control- and cathepsin-transfected cells (Figure 4C).

### The lysosomotropic detergent NDI-HCl causes cell death that is not enhanced in HeLa cells overexpressing hCath-L or hCath-B

**Figure 5:**
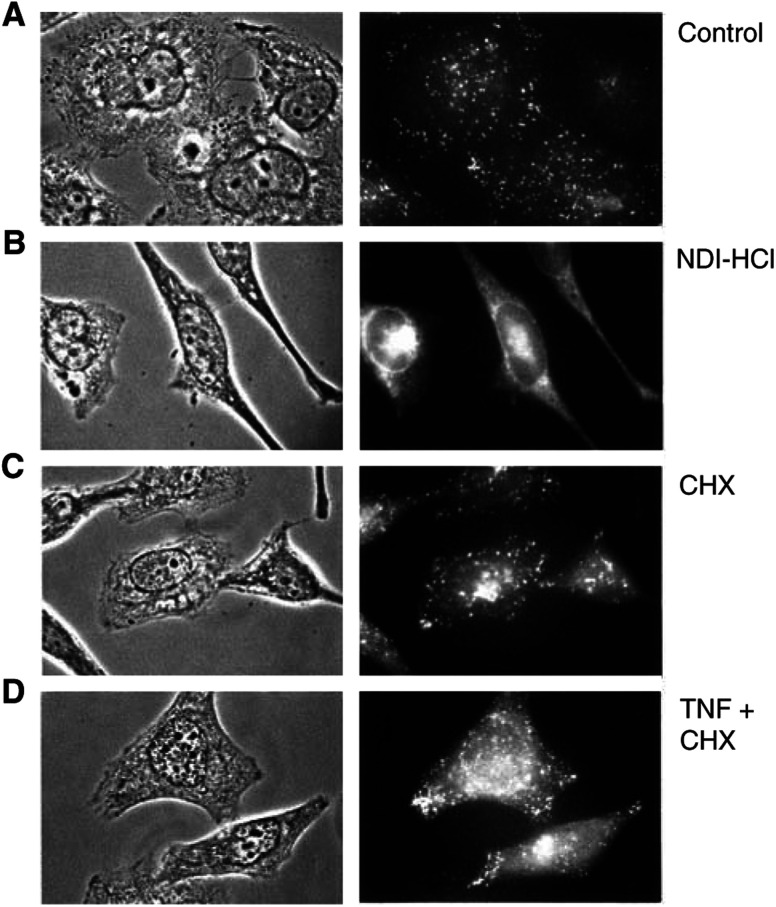
Immunostaining against endogenous Cath-L in HeLa cells treated with TNF/CHX or the lysosomotropic detergent NDI-HCl. HeLa cells were cultivated for 16 h in the absence (**A**) or presence of 100 *μ*M NDI-HCl (**B**), 2 *μ*g ml^−1^ CHX (**C**), or 10 ng ml^−1^ TNF+2 *μ*g ml^−1^ CHX (**D**) and were then fixed and immunostained using an anti-Cath-L mAb and an FITC-labelled anti-mouse mAb. Cells were microscopically inspected under a UV lamp and images were taken using a digital imaging system.

The mode of action of lysosomal proteases for apoptosis is assumed to involve the disintegration of lysosomes and the release of their content into the cytosol. Subsequently, lysosomal proteases cleave and activate proapoptotic factors such as the Bcl-2 family member Bid, which leads to the release of cytochrome *c* from the mitochondria and to the activation of caspases (Guicciardi *et al*, 2000; Turk *et al*, 2002). Consequently, we wanted to investigate whether the integrity of lysosomes in TNF-treated HeLa cells is disturbed so that elevated cathepsin levels within the lysosomes caused by overexpression can be expected to result in higher amounts of cathepsins in the cytosol. As a positive control, we treated HeLa cells with the lysosomotropic detergent *N*-dodecyl-imidazole hydrochloride (NDI-HCl), which accumulates in lysosomes and eventually damages the lysosomal membrane so that the lysosomal content is released into the cytosol (Dubowchik *et al*, 1995). Anti-Cath-L immunofluorescent staining of untreated control HeLa cells generated the expected spot-like lysosomal distribution pattern of the endogenous Cath-L (Figure 5A

). The treatment of HeLa cells with TNF resulted in a considerable number of cells with a decrease of staining intensity of discrete lysosomal spots and an apparent partial redistribution of Cath-L from those spot-like structures to a more evenly distributed pattern as shown in Figure 5D. The application of NDI-HCl to HeLa cells produced a uniform staining of Cath-L within still intact cells, indicating extensive lysosomal rupture and release of its content into the cytosol (Figure 5B).

**Figure 6:**
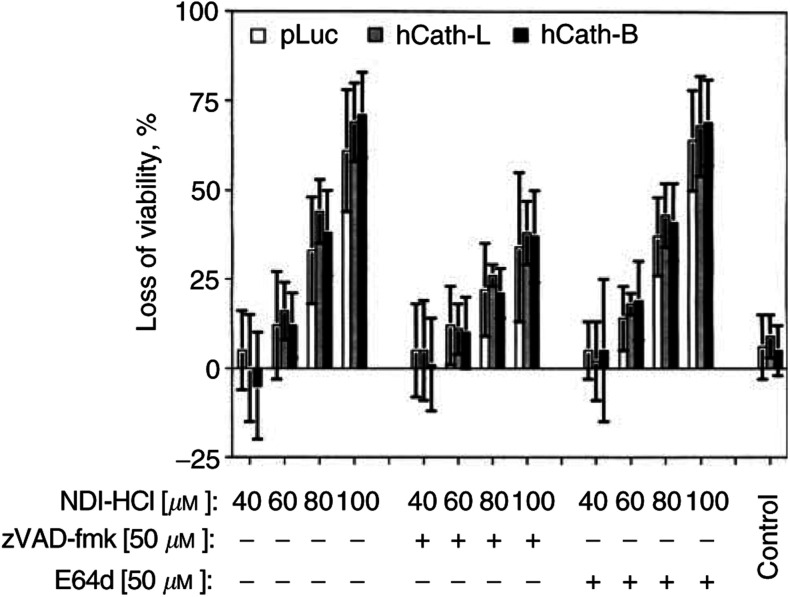
Cell death induced by NDI-HCl is not enhanced by overexpression of hCath-L or hCath-B. HeLa cells were transfected with pLuc, hCath-L, or hCath-B expression vectors; 24 h later, equal cell numbers were reseeded in wells of a 96-well plate and again 24 h later were incubated in the absence or presence of the indicated amounts of NDI-HCl in the absence or presence of either 50 *μ*M zVAD-fmk or 50 *μ*M E64d. After 22 h of treatment, cells were analysed by MTT assay as described in Materials and Methods. Transfection efficiency was determined by pEGFP cotransfection and was >50%. The data shown are the means of four independent experiments in which each data point was measured in hexaplicate.

We observed that incubation with NDI-HCl led to morphological changes resembling apoptosis. Subsequent FACS analysis confirmed apoptosis induced by NDI-HCl (not shown). We then asked whether this cell death can be enhanced by overexpression of hCath-L or hCath-B. For these measurements, we used the MTT assay in order to also include nonapoptotic cell death. As shown in Figure 6

, expression levels of hCath-L or hCath-B did not have any significant effect on NDI-HCl-induced cell death in HeLa cells as determined by the MTT assays. Apoptosis quantification by FACS analysis also did not reveal a differential cell death induction (not shown). Cell death by NDI-HCl could be partially suppressed in the presence of the pan-caspase inhibitor zVAD-fmk, indicating caspase-dependent mechanisms. Interestingly, this type of cell death could not be inhibited in the presence of the broad specificity cysteine protease inhibitor E64d (Figure 6).

## DISCUSSION

Caspases are thought to build the central proteolytic network involved in the execution of apoptotic signalling pathways induced by virtually all kinds of death stimuli (Earnshaw *et al*, 1999; Nagata, 1999; Robertson and Orrenius, 2000). In recent years, it has been recognised that besides caspases other proteases such as cathepsins, calpains, and the proteasome also may play a role as cofactors in mediating several cell death stimuli, for example, Cath-B in TNF-induced apoptosis (Leist and Jaattela, 2001). Cath-B has been reported to be released from the lysosomes to the cytosol in response to TNF (Guicciardi *et al*, 2000; Foghsgaard *et al*, 2001) and to contribute to apoptotic downstream events such as cytochrome *c* release and the activation of executioner caspases (Guicciardi *et al*, 2000; Stoka *et al*, 2001). These observations suggest a possible role for cathepsins as positive effectors of cell death pathways. This and the fact that tumours frequently contain high levels of cysteine proteases led to the hypothesis that Cath-B-like proteases may prove useful in selectively targeting tumour cells for apoptosis induction (Foghsgaard *et al*, 2001). The central objective of this study was to test the hypothesis of whether increased expression levels of Cath-L or Cath-B would enhance the sensitivity of tumour cells to TNF-mediated apoptosis. We found this not to be the case, even though inhibitor studies provided evidence for an influence of cathepsins in the TNF signalling pathway: apoptotic DNA fragmentation induced by cotreatment of HeLa and PC3 cells with TNF and CHX could be significantly suppressed by the cathepsin inhibitors E64d and CA074Me in a dose-dependent manner (Figures 1A and B), which is in line with previous reports (Guicciardi *et al*, 2000; Foghsgaard *et al*, 2001).

Interestingly, apoptosis induced by irradiating the cells with UV light could not be diminished in the presence of E64d (Figure 1C). Thus, in HeLa cells, cathepsins appear to contribute to the extrinsic receptor-mediated TNF cell death pathway, but not to the intrinsic apoptosis signalling pathways induced by UV.

The inhibitor CA074Me is usually regarded to be highly selective for Cath-B, and thus one could conclude that Cath-B is the noncaspase cysteine protease contributing to TNF-mediated apoptosis in the HeLa cell system. When we tried to verify the specific inhibitory activity of CA074Me, we realised that CA074Me not only completely blocked hCath-B enzymatic activity but also blocked hCath-L activity to almost the same extent (Figure 2B). Our observation was confirmed by a recent publication, also demonstrating that CA074Me is not a selective inhibitor of Cath-B (Montaser *et al*, 2002). Therefore, we cannot rule out the fact that besides Cath-B, CA074Me also inhibits other cysteine proteases such as Cath-L, and by this exerts its inhibitory effect.

There are several reports about strongly augmented expression levels of Cath-L and Cath-B detected in tumour cells. Frequently, the increase in cathepsin expression and activity correlated with malignant progression and metastasis possibly by direct degradation of the extracellular matrix or by activation of other proteases such as the urokinase-type plasminogen activator (Turk *et al*, 2000). Consequently, cathepsins are regarded to be tumour markers with their increased expression levels correlating with poor prognosis (Yan *et al*, 1998; Sloane, 1990; Chauhan *et al*, 1991; Sivaparvathi *et al*, 1995; Duffy, 1996; Lah and Kos, 1998). The tumour-promoting effect of cathepsins is in contrast to the observation that cathepsins such as Cath-B can mediate proapoptotic signals triggered by TNF (Foghsgaard *et al*, 2001; Guicciardi *et al*, 2001; Mathiasen *et al*, 2001), bile salts (Roberts *et al*, 1999), sphingosine (Kagedal *et al*, 2001), activation of the B-cell receptor (Katz *et al*, 2001), and L-2,5-dihydrophenylalanine (Kiso *et al*, 2001). Consequently, we wanted to address the question of whether this contrast might prove useful in developing a cancer therapy selectively targeting tumour cells for cell death, which express high levels of cathepsins (Foghsgaard *et al*, 2001). Even though this concept appears attractive, up to now no studies have been presented investigating a possible correlation between elevated cellular cathepsin levels and sensitivity to apoptotic stimuli. Since our inhibitor studies indicated a contribution of Cath-B-like cysteine proteases in TNF-induced apoptosis in HeLa cells (Figure 1), we decided to investigate whether the sensitivity of HeLa cells to the TNF signal is enhanced by increased levels of hCath-L or hCath-B. We found that the overexpression of hCath-L or hCath-B by itself does not result in overt apoptosis induction in HeLa cells, whereas overexpression of hCath-D led to apoptosis as could be expected from a previous report (Deiss *et al*, 1996). In these transient transfection experiments, we usually obtained between 40 and 60% transfection efficiency as judged by GFP cotransfection and an increase of cathepsin activity of at least 6- to 10-fold for hCath-L and 10- to 20-fold in case of hCath-B. This is comparable to the activities frequently detected in tumour tissues (Chauhan *et al*, 1991; Lah and Kos, 1998; Yan *et al*, 1998).

Consequently, under these experimental conditions we expected to be able to uncover any increase in sensitivity correlated with cathepsin overexpression. However, we did not detect a significant increase in sensitivity to TNF-induced apoptosis in HeLa cells transiently transfected with hCath-L or hCath-B (Figure 4A). Secondly, we generated HeLa cell pools stably overexpressing hCath-L or hCath-B, respectively. In this case, we could assume that the majority of cells express the corresponding cathepsin, although at a lower level than in transient expression experiments. Those stable transfected pool clones also did not show any augmented sensitivity to the TNF death signal (Figure 4B). Thirdly, we used the rat hepatoma cell line McA RH 7777 for transient expression experiments, which also did not indicate any change in sensitivity to TNF upon cathepsin overexpression (Figure 5C). We chose this additional cell line since hepatocytes have been described to be influenced by the proapoptotic activity of Cath-B (Roberts *et al*, 1999; Guicciardi *et al*, 2000, 2001).

The release of lysosomal cathepsins into the cytosol is thought to be decisive for their apoptosis induction. As shown in Figure 5, lysosomal integrity is apparently disturbed when HeLa cells are treated with TNF-*α*, as indicated by the partial redistribution of endogenous Cath-L from a lysosomal spot-like pattern to a more diffuse staining. Thus, increased expression levels of cathepsins should also be expected to result in higher amounts of cathepsin activity in the cytoplasm in response to TNF. How can it then be explained that we observe an inhibitory effect of TNF-induced apoptosis by the cathepsin inhibitors E64d and CA074Me (Figure 1), but do not see any effect upon cathepsin overexpression (Figures 4 and 5)? Firstly, it cannot be excluded that cathepsin-like proteases other than Cath-L or Cath-B are mainly responsible for the contribution to TNF-*α* apoptotic pathways, since the inhibitors E64d and apparently also CA074Me are not specific inhibitors of distinct cathepsins (Figure 2; Montaser *et al* 2002). Secondly, it is also possible that the endgenous basal levels of lysosomal Cath-L or Cath-B activity are sufficient to provide the lysosomal stimulus contributing to the proapoptotic signalling cascade for cell death execution. In this case, downstream or upstream factors other than Cath-L or Cath-B levels could be rate limiting in the execution process of apoptosis, and an increase in Cath-L or Cath-B levels would not further accelerate or enhance the signal from the TNF receptor. Supporting this, we obtained the linear relationship of a Lineweaver–Burke plot using overexpressed Cath-B and the test substrate zFR-AMC (data not shown). This indicates that cathepsin proteolysis obeys the Michaelis–Menten kinetics. Consequently, an *x*-fold increase in Cath-B levels corresponds to an *x*-fold increase of substrate turnover. Therefore, overexpression of Cath-B in our transfection experiments should correlate with an increased turnover of endogeneous Cath-B substrates.

Both possible explanations described above could be valid and would be in line with our observation that overexpression of hCath-L or hCath-B does not show any death-enhancing effect in NDI-HCl-induced cell death (Figure 6), even though it can be assumed in this case that treatment with NDI-HCl efficiently released the overexpressed cathepsins from the lysosomal compartments to the cytosol (Figure 5). We must point out that NDI-HCl-induced death behaves differently from TNF-mediated apoptosis, since NDI-HCl results in cell death that cannot be reduced by E64d, and zVAD-fmk can only partially block cell death (Figure 6), presumably due to the extreme extent of lysosomal rupture caused by this lysosomotropic detergent (Figure 5B) leading to both a caspase-dependent apoptotic mode and a caspase-independent mode of cell death.

In conclusion, Cath-B-like cysteine proteases seem to contribute to certain aspects of apoptotic signalling pathways that involve the disruption of lysosomes. Whereas the possibility appeared attractive to exploit the release of cathepsins in specifically targeting tumour cells with elevated cathepsin expression levels for cell death induction, our overexpression experiments do not support this hypothesis. This is, to our knowledge, the first report directly investigating a possible correlation between cathepsin expression levels and the sensitivity to an apoptotic stimulus, and further studies could extend these findings to other cell systems and additional cell death stimuli.
